# Effect of Protein Intake Early in Life on Kidney Volume and Blood Pressure at 11 Years of Age

**DOI:** 10.3390/nu15040874

**Published:** 2023-02-09

**Authors:** Ester Parada-Ricart, Natalia Ferre, Veronica Luque, Dariusz Gruszfeld, Kinga Gradowska, Ricardo Closa-Monasterolo, Berthold Koletzko, Veit Grote, Joaquin Escribano Subías

**Affiliations:** 1 Paediatric Nutrition and Human Development Research Unit, Universitat Rovira i Virgili, IISPV, 43201 Reus, Spain; 2 Department of Pediatrics, Hospital Universitari de Tarragona Joan XXIII, 43007 Tarragona, Spain; 3 Children’s Memorial Health Institute, 04-736 Warsaw, Poland; 4 Division of Metabolic and Nutritional Medicine, Department of Pediatrics, Dr. Von Hauner Children’s Hospital, LMU University Hospital Munich, 80337 Munich, Germany; 5 Hospital Universitari Sant Joan de Reus, 43204 Reus, Spain

**Keywords:** kidney, blood pressure, protein, child

## Abstract

High protein intake has been associated with kidney hypertrophy, which is usually reversible; however, when it occurs early in life, it could lead to cell programming with a long-lasting effect. This study aimed to assess whether higher protein ingestion early in life has a persistent effect on kidney volume at 11 years of age, as well as its influence on blood pressure. This is a secondary analysis of a randomized control trial that compared the growth of infants fed with a higher-protein formula versus those fed with a lower-protein formula, with a control group of breastfed infants. Renal ultrasound and anthropometric measurements were assessed at 6 months and 11 years of age. At 11 years, urinary protein, albumin and creatinine, and blood pressure were measured in 232 children. Feeding with a higher-protein formula was associated with a larger kidney volume (β = 8.71, 95%CI 0.09–17.33, *p* = 0.048) and higher systolic blood pressure (β = 3.43, 95%CI 0.78–6.08, *p* = 0.011) at 11 years of age. Microalbuminuria was detected in 7% of the patients, with no differences among groups (*p* = 0.56). The effect of increased protein ingestion early in life may condition kidney volume and blood pressure in later childhood.

## 1. Introduction

Kidney development is highly influenced by nutritional factors during nephrogenesis. Barker’s hypothesis proposed that a poor nutrient supply during a critical period of in utero life may “program” a permanent structural or functional change in the fetus, thereby altering the distribution of cell types, gene expression, or both [1]. In humans, the effect of intra-utero growth restriction on cardiovascular disease has been extensively documented [1]. Low nephron endowment, already determined at birth, has been associated with an increased risk of chronic kidney disease and hypertension in adulthood [2,3,4,5]. However, nutritional factors when nephrogenesis is completed, especially protein ingestion, have also been directly linked to glomerular hyperfiltration and, later, declined estimated glomerular filtration rate (eGFR) in adulthood [6].

Animal experimental models demonstrated that higher chronic protein ingestion (after nephrogenesis had ended) increased the kidney volume and glomerular filtration rate, and eventually proteinuria and glomerulosclerosis. This effect was more evident in animals with less nephron endowment [7].

In a previous study, we showed that, at 6 months of life, infants fed with a higher-protein-content formula had a significantly higher kidney volume, ratios of kidney volume to body length, and kidney volume to body surface area than those breastfed or fed with formula containing lower protein with no significant differences in eGFR [8].

The aim of the present study was to assess whether the protein-induced kidney growth early in life persisted to a later age, ten years after the end of the intervention, and if early kidney growth differences influenced blood pressure at 11 years of age.

## 2. Materials and Methods

The EU-Childhood Obesity Project (NCT00338689) is a multicenter, double-blind, randomized dietary intervention trial. Recruitment was conducted in five European countries (Germany, Poland, Italy, Belgium, and Spain) between October 2002 and July 2004. Healthy singleton term infants with birth weight > 2500 g were enrolled during the first 2 months of life. Formula-fed infants were randomly assigned to either a lower-protein-content formula (1.25 g/dL and 2.05 g/dL of protein in infant formula and follow-on formula, respectively) or a higher-protein-content formula (1.6 g/dL in infant formula and 3.2 g/dL in follow-on formula). A group of breastfed infants was recruited as a control group. Further details on the clinical trial were published elsewhere [9].

Kidney ultrasound was performed at 6 months in all centers but Italy. Infants with kidney anomalies were excluded from analysis. The results of the 6-month assessment and the possible mechanisms implicated have already been published by Escribano et al. [8] and Luque et al. [10].

At 11 years of age, a new follow-up on kidney growth was performed in Poland and Spain. 

### 2.1. Measurements

Information on demographics, socioeconomics, parent’s pre-pregnancy weight and height, the course of pregnancy, medical history, and child anthropometric data at birth was obtained from standardized parent interviews at recruitment. Self-reported data about maternal alcohol consumption and smoking during and before pregnancy were gathered in the baseline questionnaire. 

Weight was measured in underwear on a SECA 702/703 digital scale (10 g precision) and length/height were measured in a SECA 232/242 stadiometer. BMI (kg/m^2^) was calculated using the following formula: weight (kg)/height^2^ (m^2^). 

Blood pressure (mmHg) was measured with a Dinamap ProCare 100/200 digital blood pressure monitor. Blood pressure was determined in duplicate at least 15 min from arrival at the center and after at least 5 min of rest on the left arm supported by a slightly elevated horizontal support. Measurements were separated by at least 5 min. 

Kidney size was assessed by ultrasonography. Trained and blinded examiners using a linear or sector ultrasound (5–7.5 MHz) with a posterior approach in the prone position or a lateral approach in the prone position measured the longest length possible. Length, width, maximum depth in the longitudinal section (D1), and maximum depth in the transverse section (perpendicular to the hilar region) (D2) of both kidneys were measured (cm). Kidney volume was determined by the equation of an ellipsoid (kidney volume (cm^3^) = length × width × 0.5 × (D1 + D2) × 0.523). Total kidney volume (cm^3^) was calculated as the sum of the left and right kidney volumes. The relative kidney volume was computed as kidney volume/body weight (cm^3^/kg), kidney volume/BMI (cm^3^/Kg/m^2^), or kidney volume/body length (cm^3^/cm).

Urinary osmolarity (mmol/L) was measured using an osmometer. Urinary creatinine (mg/dL) was analyzed using the kinetic Jaffe’s reaction in an automated ADVIA 1650/Mega Bayer R (Leverkusen, Germany). Albuminuria and proteinuria were determined on Alinity c Analyser (Abbott Laboratories, Chicago, IL, USA) by turbidimetric immunoassay and expressed as the urinary albumin/creatinine ratio (mg/g) and urinary protein/creatinine ratio (mg/mg). Samples were analyzed in a central laboratory (Diagnostic Laboratory, The Children’s Memorial Health Institute, Warsaw, Poland).

### 2.2. Data Analysis

The distribution of the variables was assessed through graphical representation. Continuous variables are displayed as mean and standard deviation or as median and interquartile range according to their distribution. Categorical variables are described as N and percentage of the total. 

Weight, height, and BMI z-scores were estimated using World Health Organization (WHO) references [11]. Values of blood pressure were standardized as percentiles according to sex, age, and height using the references from the American Academy of Pediatrics from 2017 [12].

The Student’s *t*-test or Mann–Whitney U test was used for two-group comparisons and ANOVA was used for multiple group comparisons. Proportions were compared using the Chi-squared test. Spearman’s correlations were used to test for linear associations among continuous variables. For multiple comparisons, we applied Bonferroni correction. The association between kidney size at 11 years of age and feeding type (lower protein vs. higher protein vs. breastfeeding) was evaluated using multiple linear regression models, adjusting for potential confounders such as country, sex, maternal smoking during gestation, birth weight, and current anthropometrical parameters. To assess if the effect on kidney size at 11 years of age was mediated by the effect of nutrition on kidney size at 6 months, we conducted a causal mediation analysis using the model-based method [13].

We also assessed the association between feeding type and blood pressure at 11 years of age. We considered different models, adjusting for potential confounders such as country, sex, maternal smoking during pregnancy, birth weight, and anthropometrical parameters, as well as kidney size at 6 months and at 11 years of age. To better understand the effect of early nutrition on blood pressure at 11 years, we performed mediation analysis considering kidney size at 6 months as a possible mediator.

Statistical significance was accepted at the level of *p* < 0.05.

All statistical analyses were conducted with RStudio 2021.09.0+351 “Ghost Orchid” Release (077589bcad3467ae79f318afe8641a1899a51606, 20 September 2021) for Windows. For mediation analysis, we used the R package “mediation”.

The trial was approved by the ethics committees in each study center and written informed consent was obtained from parents or caregivers. All research was performed following the Declaration of Helsinki.

## 3. Results

Eight hundred and ten children (Germany n = 193, Belgium n = 130, Poland n = 206, and Spain n = 301) underwent a kidney ultrasound at 6 months. A kidney ultrasound was completed at 11 years in 389 children (only children from Poland n = 83 and Spain n = 174). After excluding children with kidney anomalies, 232 children (115 male and 117 female) underwent a kidney ultrasound at both 6 months and 11 years and were included in the analysis (Spain n = 166 and Poland n = 66). The baseline and follow-up characteristics according to feeding group are summarized in Table 1. The number of mothers who smoked during pregnancy was significantly lower in the breastfeeding group compared with those fed formula in the first year of life.

**Figure 1:**
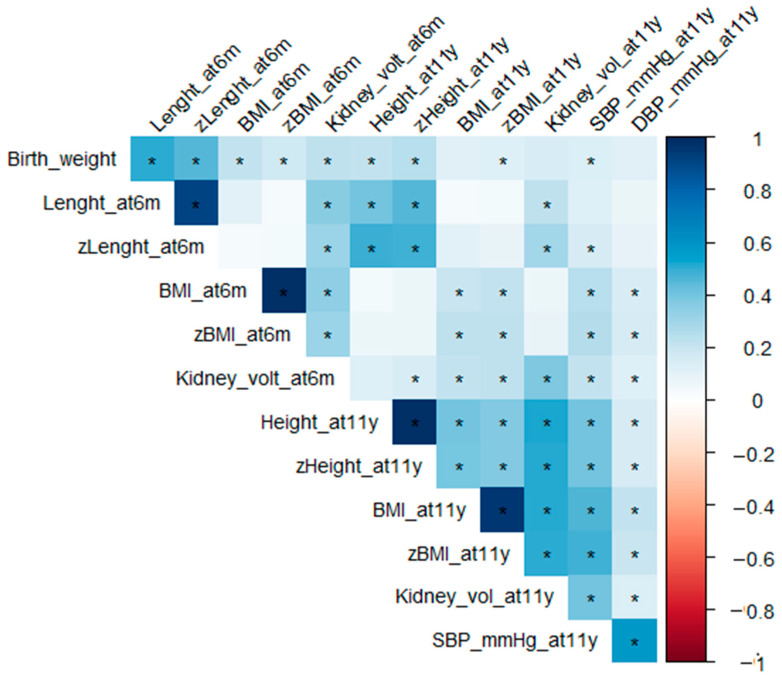
Correlation between variables. Spearman’s correlation coefficients among variables. * *p* < 0.05.

The correlation between variables is shown in Figure 1.

### 3.1. Anthropometry

Weight at birth was similar in the three groups. At 6 months, infants fed with the higher-protein formula were significantly heavier and had a higher BMI than breastfed infants. Although children fed with the higher-protein formula tended to have a higher weight and BMI at 11 years, these differences were not statistically significant in this subgroup (Table 1).

### 3.2. Kidney Size

At 6 months of age, kidney length and volume were significantly higher in the group of children fed with the higher-protein formula compared with breastfed children or those fed with the lower-protein formula. At 11 years, kidney length was larger in the higher-protein group, but volume, either absolute or adjusted for length, weight, or BMI, was similar in the three groups in the univariate analysis (Table 2). 

To analyze the relationship between kidney volume at 11 years and feeding early in life, we conducted a linear regression analysis considering potential factors that could contribute to the determination of kidney volume (sex, birth weight, maternal smoking during pregnancy, feeding early in life, height at 11 years of age, and country). Total kidney volume at 11 years was associated with height at the same age, maternal smoking during pregnancy, and being fed with the higher-protein formula during the first year (Table 3). In our cohort of children born at term with normal weight, kidney volume at 11 years was not associated with birth weight. When we included in the model kidney volume at 6 months, feeding with the higher-protein formula was not significantly associated with kidney volume at 11 years (Supplementary File).

We conducted a mediation analysis to determine if kidney volume at 6 months could be a mediator of the effect of feeding intervention in infancy on kidney volume at 11 years. The results indicate that kidney volume at 6 months significantly mediates the association of the protein formula content with a higher kidney volume at 11 years of age (HP vs. LP +6.74 cm^3^/kg, 95%CI 3.20–10.79; *p* < 0.001).

### 3.3. Microalbuminuria and Proteinuria at 11 Years

We obtained data on albuminuria and proteinuria in 200 out of the 232 children. Only one child had protein/creatinine above 0.2 mg/mg at 11 years of age (protein/creatinine of 0.26 mg/mg and albumin/creatinine of 243 mg/g). This child belonged to the group of breastfed infants and had a normal BMI (zBMI −0.54) and normal blood pressure at 11 years. Sixteen children (7%) had urine albumin/creatinine >30 mg/g: six in the lower-protein formula group, seven in the higher-protein formula group, and three in the breastfed children (*p* = 0.56).

### 3.4. Blood Pressure

At 11 years of age, children fed with the higher-protein formula had a higher systolic blood pressure percentile compared with the other two groups, but this difference was not statistically significant if we applied Bonferroni correction (higher protein vs. lower protein *p* = 0.042). No differences were found in diastolic blood pressure (Table 1). There was a direct significant correlation between total kidney volume at 11 years and systolic blood pressure at 11 years (R = 0.402, *p* < 0.01). Multivariate analysis showed a significant effect of feeding type on systolic blood pressure at 11 years of age, even when sex, country, birth weight, maternal smoking during pregnancy, and current anthropometrical variables were included in the model ((a) in Table 4). Neither kidney volume at 6 months nor kidney volume at 11 years was associated with systolic blood pressure at 11 years of age in the linear regression models ((b) and (c) in Table 4). As expected, height and BMI played a significant role in the prediction of systolic blood pressure at this age. At 11 years, 22 children (9.4%) had systolic blood pressure ≥95th percentile: six from the breastfed group and eight from each of the formula-fed groups.

**Figure 2:**
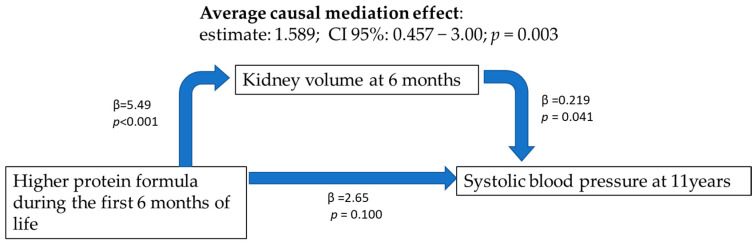
Mediation analysis on the effect of protein ingestion during the first months of life on systolic blood pressure at 11 years.

In our cohort, at 11 years of age, protein ingestion during the first year of life did not have a significant effect on BMI at 11 years in the multivariate analysis, so BMI cannot be considered a mediator of the effect of early protein ingestion on blood pressure at 11 years. Kidney volume at 6 months of age was significantly associated with protein ingestion during the first year of life. In the mediation analysis, the protein supply had a statistically significant indirect effect on SBP at 11 years, partially mediated by kidney volume at 6 months (Figure 2).

## 4. Discussion

The present work shows that early protein intake affects early kidney volume, which in turn affects blood pressure at a later school age.

### 4.1. Kidney Volume

In a previous study, we observed that a higher protein content in infant formula induced a larger kidney volume at 6 months compared with infants who were breastfed or those fed with a lower-protein-content formula [8]. It was not known at that time whether such effects would be reversible or permanent. The follow-up of this cohort until 11 years of age showed no significant differences in kidney volume when we compared the group of breastfed children with the children fed with the lower- or higher-protein formula in the univariate analysis. Nevertheless, the results of the multivariate-adjusted analyses and mediation models suggest that, indeed, there was a long-lasting association between feeding with the higher-protein-content formula and kidney volume at 11 years, and this was significantly mediated by kidney volume at 6 months of age.

There are no studies assessing the effect of protein ingestion early in life on kidney volume, but there are some studies reporting the effect of breastfeeding versus formula-feeding on kidney volume. These studies report contradictory results: Schmidt et al. reported that 3-month-old infants who were formula-fed had bigger kidneys than infants who were exclusively breastfed, but this effect was found to be reversible at the age of 18 months [14]. On the contrary, in the Generation R cohort, never breastfed children had smaller kidneys compared with ever-breastfed children even after adjusting for potential confounders at 6 years of age [15].

In the Generation R cohort, Bakker et al. [16] assessed the associations of fetal and early childhood growth characteristics with kidney volume: second- and third-trimester fetal weights, as well as gestational-age-adjusted birth weight and weight, at the age of 6 months were all independently associated with a larger combined kidney volume at 6 years, suggesting that both fetal life and early infancy may be critical periods for the development of kidney size.

One surprising result was the positive association between maternal smoking during pregnancy and kidney volume at 11 years of age. Maternal smoking during pregnancy has been linked to intrauterine growth restriction in epidemiological studies and to low nephron endowment in animal experimental studies [17,18]. The effect of tobacco exposure during pregnancy on kidney volume in humans is not clear: Smidt et al. in South Africa found no differences in kidney volume at 5 years of age in children exposed to maternal smoking during pregnancy compared with non-exposed children, although exposed children had a lower body weight, body length, and kidney length [19]. In the Generation R cohort, total fetal kidney volume was larger in the offspring of mothers who smoked less than five cigarettes per day and became smaller with increasing numbers of cigarettes smoked per day, but no significant associations were observed at 2 years of age [20]. The effect of maternal smoking during pregnancy, which could reduce nephron endowment, together with a higher protein intake during the first months of life (which could induce higher nephron hypertrophy in children who actually have less nephron endowment), might be a possible explanation for our findings.

Low nephron endowment is linked to worse cardiovascular and kidney outcomes, as demonstrated in animal and epidemiologic studies [3]. On the other hand, glomerular hypertrophy due to overload has also been shown to have a deleterious effect on glomerular sclerosis and apoptosis in the long run [21]. Higher protein ingestion is one of the factors that could contribute to that overload. The difficulty in approximating, in the clinical setting, whether kidney size is a subsidiary marker of nephron endowment (i.e., bigger size, better cardiovascular and kidney function outcomes) or a marker of glomerular hypertrophy, and will subsequently be an indicator of worse outcomes, is a critical aspect to be solved.

### 4.2. Blood Pressure

Systolic blood pressure at 11 years in our cohort was positively associated with maternal smoking during pregnancy and feeding with the higher-protein formula. These findings support the hypothesis that early developmental and environmental factors have a principal role in future health, as pointed out by the Developmental Origins of Health and Disease hypothesis [1].

The positive correlation between kidney volume at 11 years and systolic blood pressure was unexpected. In 1988, Brenner suggested that a congenital deficiency in glomerular filtration surface area contributes to a limited ability to excrete a sodium load, leading to volume-dependent systemic hypertension. Systemic hypertension will generate hyperfiltration and glomerular capillary hypertension, which will lead eventually to glomerular sclerosis, decreasing, even further, the glomerular filtration area, and thus aggravating hypertension and susceptibility to renal failure [22]. Adult hypertensive patients were found to have fewer glomeruli than their normotensive counterparts in clinical samples [23], and there is evidence that low nephron endowment is a risk factor for developing hypertension and chronic kidney disease [3,4]. Nevertheless, the results in the paediatric population are somewhat contradictory to these observations in adults. Several authors have found a positive correlation between kidney size and cardiovascular risk factors: Lizarraga-Mollinedo et al. observed an association between renal length and renal volume with carotid intima-media thickness and systolic blood pressure in prepuberal children. This association was higher in obese children [24]. Gurusinghe published that the 24-h systolic blood pressure index was positively correlated with the body-surface-area-adjusted total renal volume in children 4–20 years of age. Other authors did not find any association between kidney volume and blood pressure; however, children with smaller kidneys had higher creatinine and cystatin C blood levels, leading to a lower estimated GFR [25].

To date, no clinical accessible method exists to measure the nephron number, so we cannot be certain whether the higher kidney volume associated with higher protein ingestion in the first months of life is due to compensatory hypertrophy and will eventually lead to glomerular sclerosis and the development of hypertension and chronic kidney disease in adult life.

An interesting study conducted in rats demonstrated that early overfeeding (protein and calories) leads to a higher body mass index, kidney weight, glomeruli number (nephrogenesis continues after birth in rats), and blood pressure in adult rats, but also a higher number and larger distribution of senescent cells in the kidneys at weaning [21]. Because, in humans, nephrogenesis ends at 36 weeks of gestational age, the effect of higher protein ingestion early in life on kidney volume and blood pressure at 11 years of age cannot be explained by an increase in the glomeruli number. A future investigation should be conducted to elucidate whether a higher kidney volume at early ages (not due to a higher glomeruli number) would be associated with worse cardiovascular outcomes. In our cohort, the effect of higher protein ingestion during the first months of life on systolic blood pressure was partially mediated by kidney volume at 6 months, supporting the early programming hypothesis.

The main limitations in our study are the high rate of loss to follow-up at 11 years and the lack of data on creatinine or cystatin to assess glomerular function. Moreover, blood pressure was measured in only one appointment, so overestimation of blood pressure values or white coat hypertension cannot be ruled out.

## 5. Conclusions

Increased protein ingestion in the first months of life is associated with a higher kidney volume at 6 months of age, and this effect is still present at 11 years of age. Children fed with a higher-protein formula had higher systolic blood pressure at 11 years of age.

## Figures and Tables

**Table 1:** Population characteristics at baseline as well as anthropometric measurements and blood pressure (mean (SD)) according to feeding group in 232 children who underwent a kidney ultrasound at 6 months and 11 years of age.

	Lower Protein N = 83	Higher Protein N = 80	Breastfeeding N = 69	*p*. Overall
Country (Poland/Spain)	22/61	20/60	24/45	0.371
Sex (female) ^a^	39 (47.0)	39 (49.8)	39 (56.5)	0.470
Birth weight (kg)	3.25 (0.33)	3.20 (0.34)	3.27 (0.36)	0.417
Smoking during preg ^a^	34 (40.9)	18 (22.5)	7 (10.1)	**<0.001**
Weight at 6 m (kg)	7.74 (0.86)	7.89 (0.83) **	7.43 (0.86)	**0.004**
Length at 6 m (cm)	67.4 (2.04)	67.4 (2.40)	66.6 (2.63)	0.080
BMI at 6 m (kg/m^2^)	17.0 (1.39)	17.4 (1.36) **	16.7 (1.34)	**0.013**
Weight z-score at 6 m	0.09 (0.92)	0.27 (0.88) **	−0.20 (0.91)	**0.007**
Length z-score at 6 m	0.33 (0.90)	0.34 (1.04)	0.06 (1.10)	0.164
BMI z-score at 6 m	−0.14 (0.94)	0.11 (0.91) *	−0.31 (0.91)	**0.020**
Weight at 11 years (kg)	41.3 (9.6)	42.4 (11.6)	40.7 (10.4)	0.621
Height at 11 years (cm)	147 (6)	147 (7)	148 (8)	0.741
BMI at 11 years (kg/m^2^)	19.0 (3.5)	19.4 (4.0)	18.5 (3.3)	0.322
Height z-score at 11 years	0.35 (0.90)	0.39 (1.07)	0.47 (1.18)	0.788
BMI z-score at 11 years	0.50 (1.25)	0.61 (1.25)	0.30 (1.20)	0.309
SBP at 11 years (mm Hg)	105 (10.6)	108 (9.28)	105 (10.3)	0.137
SBP percentile at 11 years	54.7 (29.5)	65.3 (24.8) ^†^	57.0 (28.7)	**0.042**
DBP at 11 years (mm Hg)	58.0 (6.4)	58.6 (6.2)	57.6 (6.7)	0.609
DBP percentile at 11 years	36.9 (6.9)	38.8 (19.6)	35.2 (19.6)	0.532

For continuous variables, mean (SD) for categorical variables ^a^, N (%). *p*-values between specified groups: * *p* < 0.05 and ** *p* < 0.01 vs. breastfeeding. ^†^ *p* < 0.05 vs. low protein.

**Table 2:** Kidney measurements at 6 months and 11 years of age.

	Lower Protein N = 83	Higher Protein N = 80	Breastfeeding N = 69
Left kidney length at 6 m (cm)	5.44 (0.45)	5.65 (0.46) **^,†^	5.30 (0.41)
Right kidney length at 6 m (cm)	5.41 (0.45) *	5.61 (0.41) **^,†^	5.23 (0.41)
Left kidney volume at 6 m (cm^3^)	18.6 (4.7)	21.2 (5.1) **^,†^	18.0 (4.2)
Right kidney volume at 6 m (cm^3^)	18.7 (4.9)	21.2 (5.1) **^,†^	17.5 (4.5)
Total kidney volume at 6 m (cm^3^)	37.4 (8.9)	42.4 (9.3) **^,†^	35.6 (7.8)
Left kidney length at 11 years (cm)	9.11 (0.68)	9.23 (0.63) *	8.95 (0.83)
Right kidney length at 11 years (cm)	8.92 (0.83)	9.14 (0.75) **	8.73 (0.84)
Left kidney volume at 11 years (cm^3^)	77.5 (16.2)	80.7 (17.9)	77.0 (18.4)
Right kidney volume at 11 years (cm^3^)	73.3 (22.0)	77.2 (16.6)	72.3 (14.2)
Total kidney volume at 11 years (cm^3^)	151 (34.7)	158 (31.9)	149 (30.1)
Total kidney volume/weight at 11 years (cm^3^/kg)	3.75 (0.86)	3.84 (0.67)	3.75 (0.67)
Total kidney volume/height at 11 years (cm^3^/cm)	1.02 (0.22)	1.07 (0.19)	1.01 (0.18)
Total kidney volume/BMI at 11 years (cm^3^/kg/m^2^)	8.08 (1.94)	8.26 (1.29)	8.17 (1.47)

*p*-values: * *p* < 0.05 and ** *p* < 0.01 vs. breastfeeding. ^†^ *p* < 0.05 vs. low protein.

**Table 3:** Linear regression models to predict kidney volume (cm^3^) at 11 years of age.

	Total Kidney Volume at 11y		
Predictors	*β*	CI	*p*
(Intercept)	−221.65	−300.20–−143.11	<0.001
Country (Spain)	0.30	−7.80–8.39	0.942
Sex (female)	4.97	−2.51–12.44	0.192
Birth weight (kg)	8.23	−3.04–19.51	0.152
Smoking during pregnancy (yes)	9.58	0.96–18.21	**0.030**
Feeding type/intervention first year Higher protein formula	8.71	0.09–17.33	**0.048**
Feeding: breastfeeding	−1.33	−10.52–7.87	0.777
Height at 11 years (cm)	2.31	1.79–2.83	**<0.001**
R^2^/R^2^ adjusted	0.315/0.294		

CI: 95% confidence interval.

**Table 4:** Linear regression models to predict systolic blood pressure at 11 years of age.

	SBP at 11 Years (a)			SBP at 11 Years (b)			SBP at 11 Years (c)		
Predictors	*β*	CI	*p*	*β*	CI	*p*	*β*	CI	*p*
(Intercept)	91.53	79.19–103.87	**<0.001**	90.28	77.67–102.88	**<0.001**	88.47	75.05–101.88	**<0.001**
Country (Spain)	1.85	−0.64–4.33	0.144	1.86	−0.62–4.35	0.141	1.87	−0.61–4.35	0.139
Sex (female)	0.75	−1.53–3.03	0.517	0.88	−1.41–3.17	0.450	0.57	−1.73–2.86	0.627
Smoke during pregnancy	4.04	1.36–6.71	**0.003**	3.92	1.24–6.60	**0.004**	3.88	1.20–6.57	**0.005**
Birth weight	2.40	−1.10–5.89	0.178	2.03	−1.55–5.61	0.265	2.28	−1.22–5.78	0.201
Feeding									
LP formula	baseline								
HP formula	3.43	0.78–6.08	**0.011**	3.06	0.30–5.82	**0.030**	3.25	0.58–5.92	**0.017**
Breastfeeding	1.96	−0.88–4.81	0.175	2.05	−0.80–4.90	0.157	1.98	−0.86–4.82	0.171
BMI z-score at 11 years	2.87	1.89–3.84	**<0.001**	2.79	1.81–3.78	**<0.001**	2.65	1.61–3.69	**<0.001**
Height z-score at 11 years	2.52	1.36–3.67	**<0.001**	2.49	1.33–3.65	**<0.001**	2.22	0.95–3.48	**0.001**
Kidney volume at 6 m				0.07	−0.07–0.20	0.335			
Kidney volume at 11 years							0.03	−0.02–0.07	0.253
Observations	229			229			229		
R^2^/R^2^ adjusted	0.341/0.317			0.343/0.316			0.344/0.318		

SBP: systolic blood pressure, CI: 95% confidence interval.

## Data Availability

The datasets analysed during the current study will be available from the corresponding author upon reasonable request.

## Supplementary Materials

The following are available online at https://www.mdpi.com/article/10.3390/nu15040874/s1, Table S1: Linear regression models to predict kidney volume at 11 years of age, Table S2: Mediation analysis for kidney volume at 11 years.
