# Supplementary material for: Effect of Protein Intake Early in Life on Kidney Volume and Blood Pressure at 11 Years of Age
Source: Nutrients. 2023 Feb 9;15(4):874. doi: 10.3390/nu15040874 (PMC9961192; doi:10.3390/nu15040874)
Supplement: Supplementary file 1 [file nutrients-15-00874-s001.zip › nutrients-2088316-supplementary.pdf]

Table S1. Linear regression models to predict kidney volume at 11 years of age.

	Total kidney volume at 11years			Total kidney volume at 11years		
**Predictors**	**Estimates**	**CI**	**p**	**Estimates**	**CI**	**p**
(Intercept)	-221.65	-300.20 – -143.11	<0.001	-221.18	-294.79 – -147.56	<0.001
Country (Spain)	0.30	-7.80 – 8.39	0.942	0.34	-7.25 – 7.93	0.930
Gender (female)	4.97	-2.51 – 12.44	0.192	7.30	0.25 – 14.35	**0.043**
Birth weight (kg)	8.23	-3.04 – 19.51	0.152	1.13	-9.73 – 11.98	0.838
Smoke during pregnancy	9.58	0.96 – 18.21	**0.030**	7.03	-1.10 – 15.16	0.090
Feeding: higher protein	8.71	0.09 – 17.33	**0.048**	1.99	-6.42 – 10.40	0.642
Feeding: breastfeeding	-1.33	-10.52 – 7.87	0.777	0.03	-8.60 – 8.66	0.994
Kidney volume at 6m (cm^3)				1.16	0.76 – 1.56	**<0.001**
Height at 11y (cm)	2.31	1.79 – 2.83	**<0.001**	2.17	1.68 – 2.66	**<0.001**
R^2 / R^2 adjusted	0.315 / 0.294			0.401 / 0.380		

Table S2. Mediation analysis for kidney volume at 11 years

	Estimate	95% CI Lower	95% CI Upper	p-value
ACME	8.038	4.253	12.99	>0.001
ADE	1.929	-6.856	10.77	0.648
Total Effect	9.967	1.177	18.81	0.024
Prop. Mediated	0.784	0.321	3.91	0.024

ACME: Average causal mediation effects; ADE: Average direct effects
